# Possible autonomy: Finitude, vulnerability, and the ethical duty of listening

**DOI:** 10.1017/S1478951526102375

**Published:** 2026-04-20

**Authors:** João Carlos Geber-Júnior

**Affiliations:** 1 Palliative Care Team, Hospital Samaritano Higienópolis, São Paulo, SP, Brazil; 2 Hospital das Clínicas, Faculdade de Medicina, Universidade de São Paulo (HCFMUSP), São Paulo, Brazil

## Introduction

Autonomy occupies a central place in biomedical ethics and continues to guide clinical decision-making in serious illness. Together with beneficence, non-maleficence, and justice, it is often presented as a stable and universally applicable principle capable of guiding ethical practice across clinical contexts.

In palliative care, however, these principles are persistently strained by vulnerability, dependency, uncertainty, and the proximity of death. Among them, autonomy may be the most fragile. Although grounded in the promise of freedom and self-determination, it often falters when confronted with the lived realities of advanced illness.

At the bedside, autonomy rarely appears as the clear expression of a stable and fully articulated preference. More often, it emerges hesitantly within conversations shaped by uncertainty, fear, relational bonds, and the gradual recognition of finitude.

Over the past two decades, scholarship in palliative care has increasingly questioned whether autonomy, as traditionally conceived, adequately captures the moral complexity of end-of-life care (Borgstrom and Walter 2015; Broom et al. 2013). Clinical encounters near the end of life rarely present autonomous choice as a discrete, rational act. Instead, decisions unfold gradually through dialogue, emotional containment, and relational trust.

This essay argues that autonomy in palliative care is best understood not as an abstract individual capacity, but as a situated, relational, and ethically mediated practice. What emerges in clinical practice is not absolute autonomy, but what might be called *possible autonomy* – an autonomy constructed through listening, presence, and sustained ethical attention to context.

By introducing the concept of *possible autonomy*, this essay proposes a conceptual framework for understanding how autonomy becomes attainable within relationships of care under conditions of vulnerability, uncertainty, and finitude.

## Defining possible autonomy

Possible autonomy refers to the fragile and context-dependent form of agency that emerges under conditions of serious illness. Rather than assuming that patients always possess a stable and fully articulated capacity for self-determination, this concept recognizes that autonomy often develops gradually within relationships of care.

In this sense, *possible autonomy* can be understood as the form of agency that becomes attainable when ethical listening and relational care sustain the conditions for meaningful choice.

Under conditions of vulnerability, decisional capacity is rarely exercised in isolation. Patients frequently rely on clinicians and family members to interpret information, translate uncertainty, and reconstruct meaning as illness progresses. Autonomy therefore becomes less an expression of independent will than a relational achievement that unfolds through trust, dialogue, and recognition.

In theoretical terms, *possible autonomy* may be defined as the form of agency that becomes attainable in situations where illness, vulnerability, and uncertainty destabilize the conditions required for classical autonomy. Rather than assuming stable independence, possible autonomy emerges when relational care, ethical listening, and interpretive dialogue sustain the patient’s capacity to participate meaningfully in decisions about their own life.

Understanding autonomy in this way does not weaken respect for persons. On the contrary, it expands ethical responsibility by acknowledging that the conditions for meaningful choice must themselves be cultivated within clinical encounters.

## Autonomy at the bedside: Decisions under vulnerability

Decision-making in palliative care frequently involves choices that directly affect the body and the experience of time: initiating or withholding artificial nutrition, continuing or limiting ventilatory support, or redefining goals of care toward comfort.

Such decisions rarely occur through linear exchanges of information. Rather, they unfold within conversations marked by silence, ambivalence, emotional overload, and shifting expectations, often shaped by how time and prognosis are experienced and communicated in clinical practice (Geber-Junior and Forte 2025).

Empirical and clinical scholarship suggests that patients and families experience decision-making as an inherently relational process, particularly in advanced illness (Bernacki and Block 2014). Preferences are not static expressions of will; they evolve through dialogue, trust, and emotional containment.

In this context, autonomy cannot be reduced to the mere articulation of choice. To focus exclusively on expressed preferences risks ignoring the conditions under which those preferences are formed, revised, or even suspended. Respecting autonomy therefore requires attention not only to what is decided, but also to how decisions become possible.

## From individual autonomy to relational autonomy

Philosophical and clinical critiques have long highlighted the limitations of strictly individualistic accounts of autonomy, particularly in contexts of illness and dependency. Relational accounts emphasize that agency is constituted through narrative identity, moral responsibility, and interdependence (Ricoeur 1992).

Serious illness does not merely threaten biological survival; it also disrupts the narrative coherence through which individuals understand themselves and imagine their future. As Cassell argued, suffering arises when illness threatens the integrity of the person rather than merely the functioning of the body (Cassell 2004).

In such circumstances, patients frequently confront profound questions of meaning, dignity, and identity that shape how suffering is experienced and how decisions are articulated. The search for meaning becomes a central dimension of care in serious illness, influencing how individuals interpret their situation and respond to medical choices (Breitbart 2017a).

When illness destabilizes both identity and meaning, autonomy can no longer be understood as a purely individual capacity exercised in isolation. Instead, it becomes something fragile that must be sustained within relationships of care.

Previous work has suggested that strict adherence to autonomy as an abstract principle may inadvertently legitimize abandonment rather than respect (Geber-Junior 2025).

## Scarcity, trust, and constrained choice

The ethical challenges surrounding autonomy become even more pronounced in health-care systems marked by structural scarcity. In publicly funded systems, the language of informed choice frequently collides with limited resources, delayed access, and profound social inequalities.

In such contexts, consent may function less as evidence of full understanding than as an expression of trust in clinicians (Davies et al. 2019).

When options are structurally limited, autonomy risks becoming a rhetorical gesture rather than a lived reality.

## Conclusion

**This essay has proposed the concept of *possible autonomy* as a way of understanding how agency emerges in palliative care under conditions of vulnerability, uncertainty, and finitude.** In palliative care, autonomy is not a destination to be achieved but a method of care. It represents an ongoing ethical effort to restore to patients what serious illness threatens to erode: the experience of being recognized as subjects rather than reduced to clinical problems.

Possible autonomy emerges when clinicians resist the temptation to reduce ethical practice to procedural consent and instead cultivate the relational conditions that allow patients to speak, hesitate, revise, and be heard.

In the presence of finitude, autonomy does not disappear. It becomes possible only within relationships of care that sustain the fragile space in which patients can still be recognized as authors of their own lives.
